# Ag85B DNA vaccine suppresses airway inflammation in a murine model of asthma

**DOI:** 10.1186/1465-9921-10-51

**Published:** 2009-06-16

**Authors:** Jian Wu, Jun Xu, Chuang Cai, Xinglin Gao, Li Li, Nanshan Zhong

**Affiliations:** 1 Department of Respiratory Disease, Peking University First Hospital, Beijing 100034, PR China; 2 Department of Respiratory Disease, East District, Guangdong General Hospital, Guangdong Academy of Medical Science, Guangzhou 510080, PR China; 3 Guangzhou Institute of Respiratory Disease, First Affiliated Hospital of Guangzhou Medical College, Guangzhou 510120, PR China

## Abstract

**Background:**

In allergic asthma, Th2 lymphocytes are believed to play important roles in orchestrating airway eosinophilia and inflammation. Resetting the Th1/Th2 imbalance may have a therapeutic role in asthma. The mycobacterium tuberculosis 30-kilodalton major secretory protein (antigen 85B, Ag85B) can protect animals from M. tuberculosis infection by inducing a Th1-dominant response.

**Methods:**

In this study, the Ag85B gene was cloned into pMG plasmids to yield the pMG-Ag85B plasmid. The expression of Ag85B gene in murine bronchial epithelia cells was detected by Western blotting and immunohistochemical staining after intranasal immunization with reconstructed pMG-Ag85B plasmids. The protective effect of pMG-Ag85B plasmids immunization in airway inflammation was evaluated by histological examination and bronchoalveolar lavage (BAL). IL-4 and IFN-γ levels in the BAL and supernatant from splenocyte culture were determined using ELISA kits.

**Results:**

The Ag85B gene was successfully expressed in murine bronchial epithelia cells by intranasal immunization with reconstructed pMG-Ag85B plasmids. Using a murine model of asthma induced by ovalbumin (OVA), pMG-Ag85B immunization significantly inhibited cellular infiltration across the airway epithelium with a 37% decrease in the total number of cells (9.6 ± 2.6 × 10^5^/ml vs. 15.2 ± 3.0 × 10^5^/ml, p < 0.05) and a 74% decrease in the number of eosinophils (1.4 ± 0.2 × 10^5^/ml vs. 5.4 ± 1.1 × 10^5^/ml, p < 0.01) compared with the OVA-sensitized control group. There was no difference in the number of neutrophils in BAL fluid between the pMG-Ag85B group, the OVA-sensitized control group and the empty pMG group. IL-4 production was significantly decreased in the BAL fluid (32.0 ± 7.6 pg/ml vs. 130.8 ± 32.6 pg/ml, p < 0.01) and in the splenocyte supernatant (5.1 ± 1.6 pg/ml vs. 10.1 ± 2.3 pg/ml, p < 0.05) in the pMG-Ag85B group compared with the OVA-sensitized control group, while IFN-γ production was increased in the BAL fluid (137.9 ± 25.6 pg/ml vs. 68.4 ± 15.3 pg/ml, p < 0.05) and in the splenocyte supernatant (20.1 ± 5.4 pg/ml vs. 11.3 ± 3.2 pg/ml, p < 0.05).

**Conclusion:**

In a murine model of asthma induced by OVA, intranasal immunization with pMG-Ag85B significantly reduced allergic airway inflammation with less eosinophil infiltration. This protective effect was associated with decreased IL-4 and increased IFN-γ production in the BAL fluid and in the supernatant of cultured splenocytes.

## Background

Allergic bronchial asthma is a complex syndrome characterized by airflow obstruction, bronchial hyper-responsiveness and airway inflammation [1]. Elevated levels of type 2 T cell cytokines such as IL-4, IL-5 and IL-13 are recognized as factors that initiate and accelerate allergic inflammation in asthma. These cytokines promote IgE synthesis, stimulate eosinophil growth and differentiation, and augment mucus production. In contrast, type 1 T cell cytokines such as IL-2, IFN-γ and IL-12, initiate the clearance of viruses and other intracellular organisms by activating macrophages and cytotoxic T cells. The two subgroups of helper T cells are stimulated in response to different immunogenic stimuli and cytokines, and constitute an immune regulatory loop [2,3]. An imbalance between Th1 cells and Th2 cells plays an important role in the development of asthma [4]. Previous research revealed that Th2 cells could provoke airway inflammation with the restricted influence of IFN-γ [5,6]. Therefore, a strategy of upregulating the Th1 immune response or downregulating the Th2 immune response may be valuable in the prophylaxis and management of bronchial asthma [7,8].

It has been hypothesized that the increased prevalence of atopy in developed countries may be associated with the declining prevalence of some infectious diseases such as tuberculosis [9]. Since Shirakawa [10] demonstrated an inverse association between exposure to mycobacteria and the subsequent development of atopy among Japanese school children, mycobacterium exposure and its relationship to asthma has gained increasing attention. Bacille Calmette-Guérin (BCG), a live attenuated Mycobacterium bovis, which is commonly used in many countries as a vaccine against human tuberculosis, has been shown to strongly induce a Th1-like response [11]. In a murine asthma model, intranasal administration of BCG suppressed airway eosinophilia, inflammation and airway hyper-responsiveness, and was accompanied by decreased Th2 cytokine levels in BAL fluid [6,12]. It has also been reported that the BCG vaccine had a protective effect in young children against the development of allergic symptoms [13,14]. A series of animal model studies demonstrated that various preparations of mycobacterial antigens possessed prophylactic effects on antigen induced airway inflammation [6,12,15,16].

The 30-kDa major secretory protein (Ag85B) is the most abundant protein of M. tuberculosis, and is a potent immuno-protective antigen as well as a leading drug target [17,18]. Immunization with Ag85B DNA [19-22] or purified Ag85B protein [18] induced a strong antigen-specific CD4+ T cell and IFN-γ response and protected against TB [23]. More recently, it was shown that Ag85B immunization inhibited acute phase atopic dermatitis [24]. Our previous study demonstrated that, in vitro, Ag85B could enhance the Th1 response in cultured PBMCs from mite-allergic asthma patients [25]. We hypothesized that the intranasal administration of Ag85B DNA might suppress asthmatic airway inflammation by enhancing the Th1 immune response. In this study, reconstructed pMG-Ag85B DNA was intranasally administrated into C57Bl/c mice and inhibited airway inflammation in OVA-sensitized/challenged mice.

## Methods

### Animals

C57Bl/c mice were purchased from the animal center of First Military Medical University (Guangzhou, China). All animals were maintained under specific pathogen-free conditions. Experiments were conducted following the University guidelines for the care and use of laboratory animals.

### Plasmid construction

The Ag85B gene was amplified from the plasmid pMTB30, which was kindly provided by M. A. Horwitz and G. Harth, UCLA. The 5' primer (5'-ggaggatccggcacaggtatgacagacgtgagcc-3') contained a BamH I restriction site and was annealed to nucleotides -9 to +16 relative to the A residue of the initiator methionine codon ATG. The 3' primer (5'-taagtctagattcggttgatcccgtcagccgg-3'), located downstream of the stop codon, contained a Xba I restriction site and was annealed to nucleotides +992 to +971 relative to the initiator methionine codon ATG. The gene for Ag85B was cloned into pMG plasmids (InvivoGen, San Diego, California, USA) to yield the pMG-Ag85B plasmid. The clone was sequenced by double-stranded sequencing (Sangon Scientific Co. Shanghai, China). Endotoxin-free plasmid DNA was prepared and purified with the Qiagen Endotoxin-free Plasmid Maxi Kit (Qiagen, GmbH, Hilden).

### Detection of Ag85B mRNA expression by RT-PCR

Total RNA was isolated using TRIzol reagent from mice lung tissues immunized with Ag85B DNA. First-strand cDNA synthesis and PCR were performed using standard procedures. The sequences of the forward and reverse primers of pMG-Ag85B and β-actin were as follows: Ag85B: 5'-ggaggatccggcacaggtatgacagacgtgagcc-3' and 5'-taagtctagattcggttgatcccgtcagccgg-3'; β-actin: 5'-tcatgccatcctgcgtctggacct-3' and 5'-cggactcatcgtactcctgcttg-3'.

### Detection of Ag85B protein expression by Western blotting and immunohistochemistry

The supernatant of transfected murine bronchial epithelial cells was collected and condensed. Samples (20 μg of protein) underwent electrophoresis on a SDS-PAGE gel. Proteins bands were probed with Ag85B antibodies. Ag85B standard protein (100 ng) was the positive control.

Ag85B protein expression in vivo was detected using immunohistochemistry staining. Lung tissue sections were then incubated with 3% H_2_O_2 _for 10 minutes, blocking buffer (0.1 M phosphate buffer containing 1% BSA and 10% normal goat serum) for 10 min at room temperature and the primary anti-Ag85B antibody overnight at 4°C. The monoclonal antibodies were raised in female New Zealand White rabbits against a purified 30 kDa protein (Ag85B). Anti-rabbit biotinylated antibody was added at room temperature followed by avidin-horseradish peroxidase conjugate.

### Intranasal immunization

OVA solution was made by mixing 20 μg OVA (Sigma Chemical Co., Louis, Missouri, USA) with 2 mg alum in 100 μl saline. All of the mice were anaesthetized with 50 mg/kg pentobarbital sodium. Mice in the three groups, except for normal control group, then intraperitoneally injected with 100 μl OVA solution on days 0, 7 and 14. On days 21 and 28, mice were grouped and immunized with 100 μg of endotoxin-free pMG-Ag85B plasmids, empty pMG or saline. The three groups were then intranasally administered 200 μg OVA on days 42, 43 and 44. On the following 2 days, mice were exposed to nebulized 1% OVA for 30 min. Mice sensitized and challenged with OVA, treated with saline during the resting phase served as OVA-sensitized control. Mice always treated with saline served as normal control group.

### Bronchoalveolar lavage and histopathological examination

Mice were sacrificed 24 hours after the last OVA treatment. In each group, seven animals were used for BAL fluid and another six for lung histopathological examination. After retro-orbital bleeding under anesthesia, lungs were lavaged three times with 0.8 ml PBS and the BAL fluid was collected. The supernatants were removed and stored at -20°C. Cell pellets were resuspended in 1 ml PBS and total cells were counted with a hematocytometer. For histopathological examination, the right and left lungs were sectioned from top to bottom, with four-to-five cross-sectional pieces taken from each lung.

### Splenocyte culture

Mouse spleens were harvested, minced and filtered through a fine nylon mesh. Red blood cells were removed using ACK lysing buffer (Invitrogen Life Technologies). Cells were then incubated in RPMI-1640 medium (Gibco BRL) supplemented with 10% fetal calf serum, 2 mM L-glutamine and antibiotics. Supernatants were collected after incubation for 96 hours.

### Enzyme-linked immunosorbent assay (ELISA) for cytokine production

IL-4 and IFN-γ levels in BAL fluid and the supernatant of cultured splenocytes were determined using ELISA kits (R&D Systems). The assay inter-well variances were <10% for cytokine concentrations ranging 5–10 pg/ml.

### Statistical analysis

Data are presented as means ± SD. Unpaired two-tailed Student's t-test was used to determine significant differences between groups.

## Results

### Expression of the Ag85B gene

**Figure 1:**
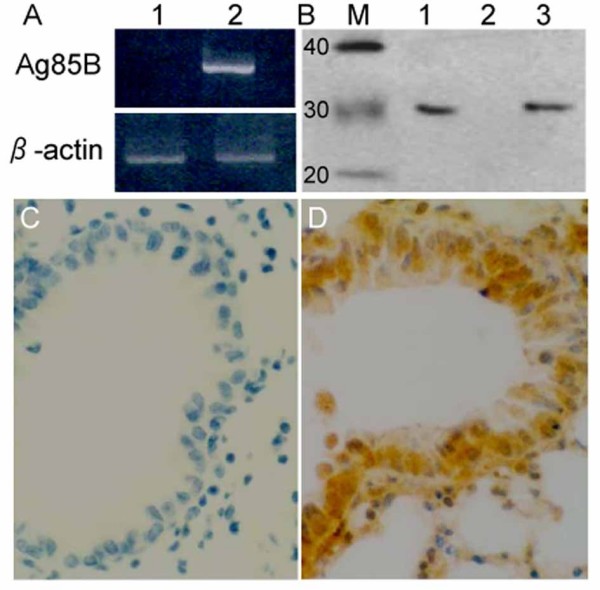
**Ag85B expression in murine bronchial epithelial cells**. A. Murine cells were transfected with pMG plasmids (Lane 1) or pMG-Ag85B plasmids (Lane 2). Ag85B mRNA (992 bp) expression was tested 36 hours after transfection by RT-PCR. B. As described in A, Western blotting was used to determine Ag85B protein expression in the supernatant of pMG-Ag85B transfected murine bronchial epithelial cells (Lane 1) and pMG transfected cells (Lane 2). The positive control was 100 ng purified Ag85B protein (Lane 3). C, D: Mice were intranasally immunized with 100 μg pMG (C) or pMG-Ag85B plasmids (D), with a booster dose 7 days after the initial immunization. Immunohistochemistry staining shows Ag85B protein expression in the lung 48 hours after the booster dose.

The Ag85B expression vector, pMG-Ag85B was constructed by inserting a 992-bp Ag85B gene into the XBal I and BamHI sites of the pMG vector. Transfection was confirmed by restriction enzyme digestion, PCR and sequential analysis (data not shown). Ag85B mRNA was detected in murine bronchial epithelial cells 36 hours after transfection with endotoxin-free pMG-Ag85B plasmids, but not in pMG plasmid-transfected cells (Fig. 1A). Ag85B protein was also detected in the supernatant of the pMG-Ag85B-transfected cells using Western blotting (Fig. 1B). We then examined Ag85B gene expression in vivo. Ag85B mRNA was detected in lung tissue 36 hours after the second intranasal immunization with pMG-Ag85B. Immunohistochemical staining revealed that the Ag85B gene was mainly expressed in bronchial epithelial cells, bronchiolar submucosa and alveolar epithelial cells (Fig. 1D).

### Immunization with pMG-Ag85B DNA protected mice from airway eosinophilic inflammation

**Figure 2:**
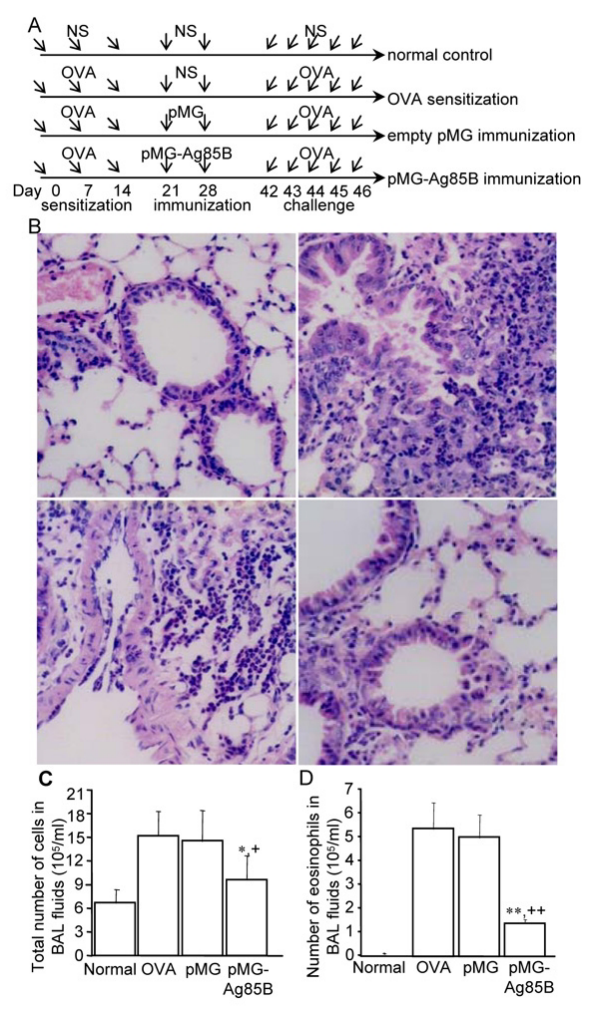
**Immunization with pMG-Ag85B inhibited inflammatory cell infiltration in the lung**. A. Timing of the sensitization, immunization and challenge (NS = normal saline). B. Lung tissue was taken 24 hours after the last OVA challenge. H&E staining of lung sections from the normal (upper left), OVA (upper right), empty pMG (lower left) and pMG-Ag85B (lower right) groups. n = 6 mice per group. C, D. BAL fluid was collected 24 hours after the last OVA challenge. The total number of cells (C) and number of eosinophils (D) were counted. Values are means ± SD for seven animals. *P < 0.05, **P < 0.01, for the pMG-Ag85B group versus the OVA-sensitized control group; +P < 0.05, ++P < 0.01, for the pMG-Ag85B group versus the pMG group (unpaired two-sided Student's t-test).

Since Ag85B was successfully expressed in vivo, we wondered whether Ag85B could protect mice from the development of asthma. We used the OVA sensitization/challenge asthma model. In this model, mice were intraperitoneally injected with high doses of OVA protein once a week for 3 weeks, rested for 4 weeks, and then challenged with OVA through the airway. These mice developed serious inflammation in the lung compared with the saline-treated mice, mimicking the pathological process of asthma. In this study, during the resting phase, mice were intranasally immunized twice with pMG-Ag85B plasmid DNA, empty pMG or saline. All mice were then challenged with 1% OVA through the airway except the saline group and lung inflammation was examined 24 hours later (Fig. 2A). In the OVA-sensitized control group, histological examination revealed shedding of the airway epithelium and swelling of the bronchiolar wall with cellular infiltration, particularly in the parabronchiolar and perivascular area (Fig. 2B, upper right). However, pMG-Ag85B immunization greatly inhibited cellular infiltration across the whole area (Fig. 2B, lower right). No inflammation was observed in the saline group (Fig. 2B, upper left). Consistent with the histological data, the total number of cells and the number of eosinophils in the BAL fluid was significantly increased in the OVA-sensitized control group compared with the saline group (Fig. 2C&2D). In the pMG-Ag85B group, OVA-induced inflammation was suppressed with a 37% decrease in the total number of cells (9.6 ± 2.6 × 10^5^/ml vs. 15.2 ± 3.0 × 10^5^/ml, p < 0.05) and a 74% decrease in the number of eosinophils (1.4 ± 0.2 × 10^5^/ml vs. 5.4 ± 1.1 × 10^5^/ml, p < 0.01) compared with the OVA-sensitized control group. There were no significant differences in the total number of cells or number of eosinophils between the empty pMG group and the OVA-sensitized control group (Fig. 2C&2D). There was no significant difference in the number of neutrophils in BAL fluid between the pMG-Ag85B group, the OVA-sensitized control group and the empty pMG group (pMG-Ag85B: 2.2 ± 0.4 × 10^5^; OVA-sensitized control: 2.5 ± 0.5 × 10^5 ^cells; pMG: 2.3 ± 0.5 × 10^5^; p > 0.05), while the number of neutrophils in BAL fluid was significantly increased in the three test groups compared with that in the normal control group (normal control group: 0.8 ± 0.1 × 10^5 ^cells; all p < 0.05)

### Cytokine production in BAL fluid and splenocytes after pMG-Ag85B immunization

Previous studies revealed an imbalance between Th1 and Th2 cells in asthma models. This phenomenon was considered an important pathogenic mechanism of asthma. We wondered whether the cytokine profile was reversed by pMG-Ag85B immunization in the asthma model during the protective process.

**Figure 3:**
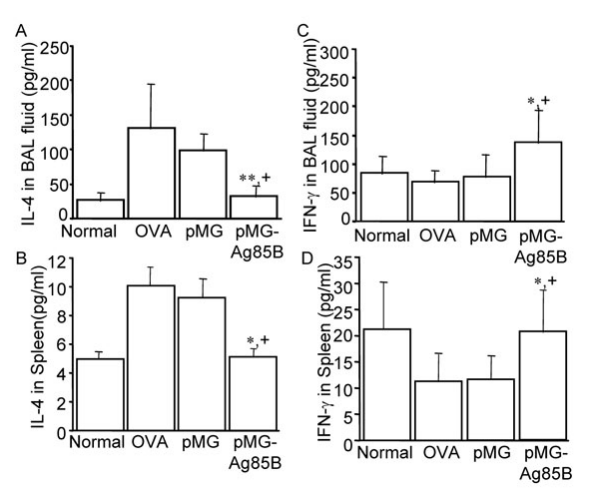
**Cytokine production in the BAL fluid and spleen after pMG-Ag85B immunization**. Mice were sensitized with OVA 3 times, and administered with pMG-Ag85B plasmid DNA, and then challenged with OVA. BAL fluid and spleens were harvested 24 hours after the last OVA challenge. IL-4 (A) and IFN-γ (C) levels in the BAL fluid were measured directly. Splenocytes were cultured and IL-4 (B) and IFN-γ (D) in the culture supernatant were measured 96 hours after incubation. Results are expressed as means ± SD for seven animals. *P < 0.05, **P < 0.01, for the pMG-Ag85B group versus the OVA-sensitized control group; +P < 0.05, ++P < 0.01, for the pMG-Ag85B group versus the pMG group.

BAL fluid was collected 24 hours after the last OVA challenge. Splenocytes were cultured and the supernatant was obtained at 96 hours. Levels of IL-4 and IFN-γ were tested using ELISAs. In the OVA-sensitized control group, IL-4 production in the BAL fluid was 5-fold higher than in the saline group (Fig. 3A) and 2-fold higher in the splenocyte supernatant (Fig. 3B). However, in the pMG-Ag85B group, IL-4 production was significantly decreased both in the BAL fluid (32.0 ± 7.6 pg/ml vs. 130.8 ± 32.6 pg/ml, p < 0.01) and in the splenocyte supernatant (5.1 ± 1.6 pg/ml vs. 10.1 ± 2.3 pg/ml, p < 0.05) compared with the OVA-sensitized control group. In addition, pMG-Ag85B immunization increased IFN-γ production both in the BAL fluid (137.9 ± 25.6 pg/ml vs.68.4 ± 15.3 pg/ml, p < 0.05) and the splenocyte supernatant (20.1 ± 5.4 pg/ml vs. 11.3 ± 3.2 pg/ml, p < 0.05) (Fig. 3C&3D).

## Discussion

Bronchial epithelial cells (BECs) are known to play an integral role in the airway defense mechanism, which involves the mucociliary system as well as mechanical barriers. BECs also interact with immune and inflammatory cells by direct adhesion as well as by humoral factors including cytokines, and may play a crucial role in mucosal immunity [26]. In the present study, the Ag85B gene was successfully expressed in murine BECs after transfection with the pMG-Ag85B plasmid. Mice with repeated OVA sensitization and aerosol challenge mimicked human allergic asthma. Intranasal administration of Ag85B DNA significantly inhibited airway eosinophilia with a 74% decrease in number of eosinophils in BAL fluid and attenuated eosinophilic airway inflammation. The inhibitory effect was associated with increased IFN-γ levels and decreased IL-4 levels in BAL fluid and in the supernatant of cultured splenocytes. These results are consistent with previous studies in which BCG was administered by the nasal route in murine allergic rhinitis [27] or in asthma models [6,12,28]. In addition, intranasal administration or direct instillation into the trachea are easier to reach [26]. They have been shown to be the most effective routes in reversing antigen-induced asthma symptoms, BAL and peribronchial eosinophilia, and BAL fluid IL-5 levels [29]. These routes were also superior to the intraperitoneal or subcutaneous routes [6]. Our data support the notion that Th2 cytokines are involved in Ag-induced allergic responses. We also provide the first in vivo evidence that an Ag85B DNA vaccine inhibits OVA-induced airway inflammation. This inhibitory effect was associated with the switch from Th2 cytokine production to Th1 cytokine production in the lung and at the systemic level. These data are in accordance with recently reported results from studies that used noninvasive mucosal exogenous gene delivery in mice models of asthma. IL-18, IL-12 and IFN-γ gene-expressing plasmids or transferred by an adenovirus vector [30-32] can prevent and reverse established allergen-induced airway hyper-reactivity, airway eosinophilia and Th2 cytokine production. Our previous in vitro study showed that the supernatant from cultured murine BECs transfected with Ag85B DNA plasmids up-regulated IFN-γ levels in peripheral blood mononuclear cells from mite-allergic asthmatic patients [25]. Therefore, our studies and other previous studies suggested that BECs are a promising target for intranasal Th1 modulator genes in the management of allergic pulmonary disease, and that intranasal administration is a safe, efficient and noninvasive mucosal route of treatment against allergic asthma [33].

A large quantity of data obtained from human and animal models demonstrated that BCG vaccine and other mycobacteria have preventive and therapeutic effects on atopic diseases such as allergic asthma [6,12-16]. But, there seems to be a discrepancy. Factors such as timing of vaccination, the route of delivery, genetic contribution and ethnicity, and dose and strain differences, could be responsible for the discrepancies that have been observed [34]. Furthermore, inoculation with BCG in humans can only be performed by intradermal administration, and may induce more adverse reactions including suppurative lymphadenitis, local abscess, and anaphylaxis during vaccination [35]. Repeated BCG injections in asthmatic patients showed no efficacy on markers of asthma severity in addition to excessive local reactions to BCG [36]. Thus, these limitations have limited the use of BCG in asthma.

Ag85B consists of a few specific molecules and is the most abundant extracellular protein expressed by Mycobacteria or BCG. In addition, it can be delivered by intranasal or intramuscular injection [19,21]. Therefore, it can be expected to be safer with a lower incidence of adverse events compared with BCG for protecting mice against TB [18] or atopic disease.

The mechanism of Ag85B immunization against TB infection is relative to the attenuation of the Th2 cell-mediated immune response and increased IFN-γ production [27]. The mechanism of Ag85B immunization against asthma is unclear. However, it might be due to increased IFN-γ production. IFN-γ was suggested to suppress pulmonary eosinophilia via the following pathways: first, by blocking IL-4, thus down-regulating the IL-12 receptor pathway and leading to development of T cells restricted to the Th1 phenotype; second, by activating highly phagocytic macrophages and preventing airway allergens from entering the submucosal sites containing the professional antigen-presenting cells and sensitized T cells [28]; and third, by inhibiting chemokines (for instance, eotaxin) and CC chemokine receptor 3 (CCR3) expression during allergic inflammation [1,29]. These are essential for eosinophil homeostasis and infiltration by Th2 cells, and thus suppress the development of an atopic phenotype. Furthermore, it is possible that Mycobacterial major secretary proteins, such as Ag85B, can generate regulatory T cells [37] and reverse allergic diseases. It has been shown that Ag85B DNA immunization can prevent and treat atopic dermatitis through the induction of Foxp3+ T regulatory (Treg) cells [24]. Several studies have shown that Mycobacterial lipoproteins [38] or mycobacterium vaccae [39] bind to dendritic cells and macrophage-bound Toll-like receptors (TLRs) and this interaction leads to the prominent synthesis of IL-12, and thus induces protective Th1 immunity with an increase in the number of Treg cells, which also controls IgE antibody production. However, it remains to be elucidated whether Ag85B triggers Treg cells in addition to eliciting strong protective Th1 immune responses. In addition, intranasal administration of the Ag85B DNA vaccine after exposure to OVA is a form of mucosal immunotherapy. The immune system in the aerodigestive mucosa maybe induce immune tolerance rather than immunostimulation and then decrease the airway eosinophilic inflammation [33]. In preclinical models, T-cell anergy, a decrease in the Th2 response, and an induction of TGF-β- and IL-10-producing regulatory T cells have been proposed to be potential mechanisms for immune tolerance through the nasal route [40,41].

In this study, we found that OVA-induced airway inflammation was inhibited after Ag85B vaccine treatment; meanwhile, Th1 cytokine production was increased while the Th2 cytokine production was decreased in the lung and spleen. However, it was unclear whether the inflammatory inhibition was due to the direct effect of the vaccine or the Th1-biased response. Moreover, the Ag85B vaccine might drive T cells to switch into Th1 cells, which subsequently suppress airway inflammation by Th1 cytokine production. To investigate the mechanism, further studies of the effect of the Ag85B vaccine are required using IFN-γ^-/- ^or IL-4^-/- ^mice. In addition, more studies of Treg cells are needed to evaluate the inhibition of eosinophil recruitment in the lung and other asthmatic symptoms, and to determine the critical roles of the Th1 and Th2 cytokines in mediating these effects.

## Conclusion

In summary, we have described a novel approach of intranasal administration of Ag85B DNA to inhibit eosinophilic airway inflammation induced by OVA sensitization. This was associated with down-regulation of Th2 cytokines and up-regulation of Th1 cytokines. Further studies are needed to investigate the effect of Ag85B DNA on bronchial hyper-responsiveness and Treg cells. Because intranasal administration of Ag85B gene is non-invasive, effective and can be easily modified, it offers a promising method for the development of DNA vaccines to asthma.

## Abbreviations

IL-4: interleukin-4; IL-5: interleukin-5; IL-13: interleukin-13; IL-12: interleukin-12; IFN-γ: interferon-γ; OVA: ovalbumin; pMG-Ag85B: encoding Ag85B gene insert into plasmid pMG; Th1: T helper-type 1; Th2: T helper-type 2; BCG: Bacille Calmette-Guérin; Treg: Regulatory T cell; Ag85B: Antigen 85B; BAL: bronchoalveolar lavage; BECs: Bronchial epithelial cells; CCR3: CC chemokine receptor 3; TLRs: Toll-like receptors; TGF-β: transforming growth factor-β; NS: normal saline.

## Competing interests

The authors declare that they have no competing interests.

## Authors' contributions

JW carried out the molecular biological, histological and immunological studies and drafted the manuscript. JX participated in the design of the study. CC helped to draft the manuscript. XG participated in the design of the study. LL carried out the ELISA. NZ conceived the study, participated in its design and coordination, and helped to draft the manuscript. All authors read and approved the final manuscript.
